# Past, Present, and Future Perspectives on Whey as a Promising Feedstock for Bioethanol Production by Yeast

**DOI:** 10.3390/jof8040395

**Published:** 2022-04-12

**Authors:** Jing Zou, Xuedong Chang

**Affiliations:** College of Food Science and Technology, Hebei Normal University of Science and Technology, Qinhuangdao 066600, China; cxdsgx@163.com

**Keywords:** whey, bioethanol, lactose, *GAL* gene, *Kluyveromyces*, *Saccharomyces cerevisiae*, metabolic engineering, optimization condition, evolutionary engineering

## Abstract

Concerns about fossil fuel depletion and the environmental effects of greenhouse gas emissions have led to widespread fermentation-based production of bioethanol from corn starch or sugarcane. However, competition for arable land with food production has led to the extensive investigation of lignocellulosic sources and waste products of the food industry as alternative sources of fermentable sugars. In particular, whey, a lactose-rich, inexpensive byproduct of dairy production, is available in stable, high quantities worldwide. This review summarizes strategies and specific factors essential for efficient lactose/whey fermentation to ethanol. In particular, we cover the most commonly used strains and approaches for developing high-performance strains that tolerate fermentation conditions. The relevant genes and regulatory systems controlling lactose utilization and sources of new genes are also discussed in detail. Moreover, this review covers the optimal conditions, various feedstocks that can be coupled with whey substrates, and enzyme supplements for increasing efficiency and yield. In addition to the historical advances in bioethanol production from whey, this review explores the future of yeast-based fermentation of lactose or whey products for beverage or fuel ethanol as a fertile research area for advanced, environmentally friendly uses of industrial waste products.

## 1. Overview of Bioethanol Production and Whey as a Feedstock

Energy security and environmental safety are two major issues currently faced by the global population that have led to elevated demand for alternative and ecologically sustainable energy sources. By some estimates, fossil fuel reserves could be exhausted within the next 40–50 years due to the rapidly increasing consumption of these non-renewable fuels [1]. More importantly, the burning of fossil fuels contributes to the emission of greenhouse gases and, consequently, global warming, which causes further climate change, rise in sea level, loss of biodiversity, and urban pollution [2,3,4]. In particular, bioethanol refers to the alcohol produced from biological sources through the fermentation of starches, sugars, cellulose, or other waste by-products of industrial food or chemical processing. Ethanol is distinct from gasoline in that it has both higher evaporation enthalpy and laminar flame speed, which results in more efficient combustion with less greenhouse gas emission. [5,6,7,8]. This higher evaporation temperature of ethanol results in higher volumetric efficiency and thus more power generated by a gasoline/ethanol blend than that provided by an equivalent unit of gasoline [9]. Specifically, bioethanol is more highly oxygenated than gasoline (i.e., 34.7% oxygen) which improves its combustion efficiency by 15%. 

First- and second-generation bioethanol can be categorized according to the feedstocks used for production. The primary raw materials for first-generation bioethanol are starch and sugars generally obtained from starchy cereal crops, especially corn, due to its global availability, relatively simple and high yield conversion, and amenability to long-term storage. The United States is the dominant producer of corn ethanol, with stated goals of 36 billion gallons of ethanol production annually by 2022 using corn and corn stover as inputs [10]. Sugar-based feedstocks include some energy crops, such as sugarcane, sugar beet, and sweet sorghum as well as sugar refinery wastes, namely cane and beet molasses [11,12,13,14]. These sugar crops offer advantages over other feedstocks through greater sugar yields and lower costs of ethanol conversion compared. However, the availability of these crops can be limited by growing season [13].

Among second-generation bioethanol, lignocellulosic biomass is the most commonly used feedstock since it represents the largest resource pool worldwide and can thus be obtained for ethanol production without competing for arable land and agricultural inputs with crops for human or livestock consumption [15,16,17]. The chief components of lignocellulosic biomass that are converted to ethanol include cellulose, hemicellulose, and lignin [16,18,19,20,21]. However, the major drawback of these feedstocks is the recalcitrance to degradation of the lignocellulosic matrix, which is comprised of covalently and hydrogen-bonded cellulose and hemicellulose polymers that are further linked to lignin in its natural state [21,22]. In addition, *Saccharomyces cerevisiae*, the most widely used ethanol-producing species in bioethanol production, has difficulty utilizing β-d-xylose and α-l-arabinose, the main pentoses in hemicellulose polymers [18,23,24,25,26]. These factors thus limit the application of lignocellulosic biomass in ethanol production until the efficiency of its degradation can be improved. 

Apart from starch materials and lignocellulosic biomass, industrial waste, especially from food production, offers a range of viable feedstock options for fuel ethanol production. Among these by-products, whey generated by the dairy industry can be reliably substituted for sugar.

**Figure 1:**
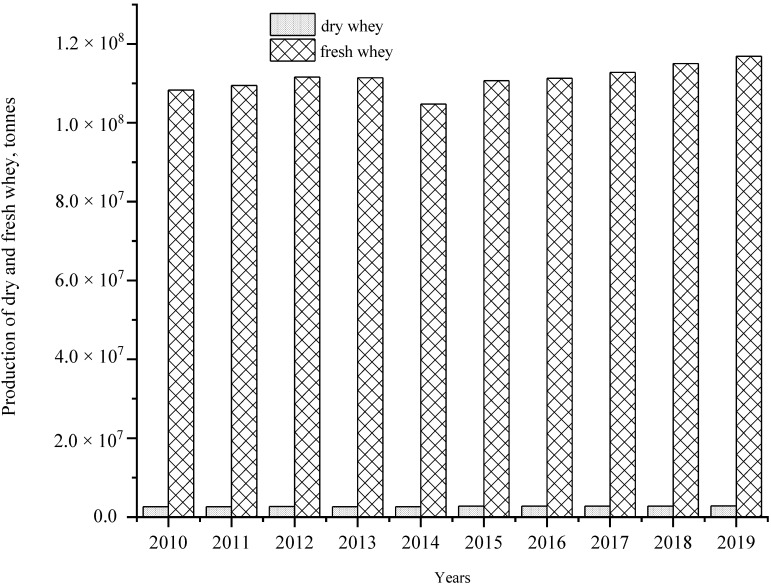
Worldwide production of dry whey and fresh whey from 2010 to 2019 (FAOSTAT, 2022).

In the process of cheese making or casein production, the water component of milk is largely separated into the whey, while the solids aggregate in curds, which are further processed. Whey can be categorized as either sweet whey, which results from casein coagulation by rennet activity, or acid whey, produced by acid coagulation of milk. The former is typical of aged cheeses, while the latter is commonly used in fresh cheese production, such as cottage cheese or ricotta [27,28]. Table 1 shows the average composition of sweet and acid wheys. In general, acid whey has a characteristically low pH, while the pH of sweet whey is around 6.5 [29]. Whey accounts for approximately 85–90% of milk used to make aged cheeses. In 2019, the FAO reported that a total of 116.9 million tons of fresh whey were produced globally, compared to 2.8 million tons of dry whey (Figure 1), with 90% of fresh whey coming from facilities in Europe and the Americas (FAOSTAT, 2022). Whey contains roughly 55% of the nutrients from milk, and it includes a lactose content of 6.8–8.5% and 0.8–2% mineral salts, depending on the production method [30,31,32,33]. 

Despite its generally high biochemical and chemical oxygen demands (BOD and COD, respectively), which are associated with environmental and health problems [34,35], in many ways, whey represents an ideal substrate for fermentation into biofuel. Moreover, whey can serve as an inexpensive resource for high-energy fuel gas production (e.g., hydrogen gas [36,37,38] or methane [39,40]) in anaerobic fermentation systems. In addition to gaseous biofuels, whey can also be used as a feedstock in the fermentative production of liquid fuels such as bioethanol [41,42], bio-butanol [43,44], or other commercially valuable chemicals (e.g., 2,3-butanediol [45,46] or organic acids such as lactic acid, caproic acid, or citric acid [47,48,49]). Despite these numerous possible uses, bioethanol production has emerged as the predominant product of industrial whey fermentation.

Therefore, whey is indeed an ideal alternative feedstock for fuel ethanol feedstock because it can provide a remarkable 6–10 million tons of lactose annually. However, despite the availability of this massive, underutilized resource, it faces challenges in being adopted at a commercial scale since *S. cerevisiae*, the most common fermentative species for transforming sugars to ethanol, lacks the enzyme required for lactose utilization and thus cannot ferment whey into ethanol without further metabolic engineering. In this paper, we review the past and future of *S. cerevisiae* and whey/lactose in bioethanol production and introduce recent advances in whey/lactose utilization for biofuel fermentation, with the broader goals of finding appropriate solutions to whey pollution and resolving the shortage in raw materials for biofuel.

## 2. The Lactose/Galactose Metabolic Pathway and Its Regulation in Fungi

### 2.1. Lactose/Galactose Consumption in Fungi

**Figure 2:**
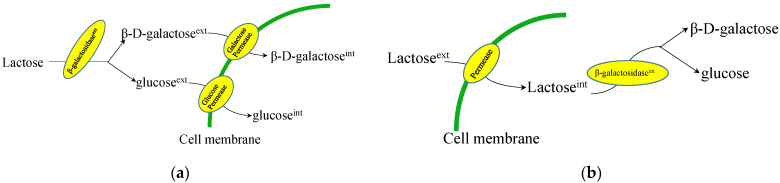
Lactose metabolism in different microorganisms. (**a**) In *Trichoderma reesei*, β-galactosidase (*bga1*) is secreted to the extracellular space, where it converts lactose into equimolar glucose and galactose monosaccharides, which are then imported into the cytoplasm. (**b**) In *Aspergillus nidulans* and *Kluyveromyces* spp., lactose is first transported into the intracellular space by lactose permease (*LAC12*) where it is catalyzed into galactose and glucose by cytosolic β-galactosidase (*LAC4*).

Lactose is a disaccharide formed from galactose and glucose and chemically defined as O-ß-d-galactopyranosyl-(1–4)-β-d-glucose. In addition to lactic acid bacteria and enterobacteria that are able to utilize lactose as a sole carbon through different transport systems [50,51,52,53], some fungi, such as *Aspergillus* [54,55,56], *Trichoderma reesei* [57,58], and members of the yeast genus *Kluyveromyces* [59,60,61] also display this metabolic capability. In these fungi, lactose is utilized through two principal mechanisms: (1) the lactose is extracellularly hydrolyzed into d-glucose and d-galactose, which are subsequently taken up by the fungi (Figure 2a); and (2) the disaccharide is first imported into the cytosol and then hydrolyzed into glucose and galactose (Figure 2b).

**Figure 3:**
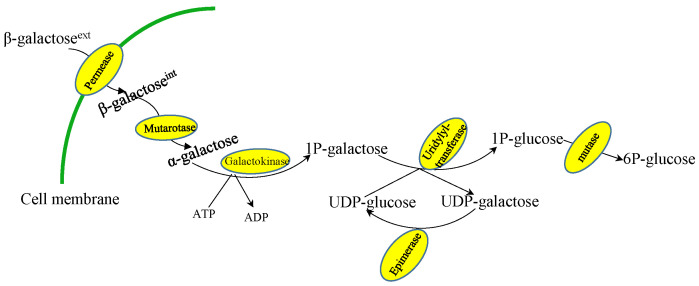
The Leloir pathway of d-galactose catabolism.

*T. reesei* uses the former pathway for lactose uptake, although the extracellular β-galactosidase (encoded by *bga1* gene) is a critical factor for lactose-induced cellulase production [62,63]. *A. nidulans* and *K. lactis*, a model microorganism of *Kluyveromyces* yeast, both utilize lactose through the latter strategy by lactose permease (encoded by *LAC12* gene)-mediated transport of the disaccharide into the intracellular compartment and subsequent hydrolysis by β-galactosidase (encoded by the *LAC4* gene) into glucose and β-d-galactose. This review does not discuss the catabolism of d-glucose in detail since several current reviews already address this topic. In most eukaryotes, α-d-galactose is converted by aldose 1-epimerase to the α-anomer before entering the Leloir pathway (Figure 3) [64,65], although filamentous fungi contain a second pathway, the oxido-reductive catabolic pathway, for α-d-galactose catabolism [55,66]. However, some strains of *K. marxianus* have been found to hydrolyze lactose outside of the cell (Figure 2a) [67,68].

Unlike *K. lactis*, *S. cerevisiae* lacks the gene for β-galactosidase synthesis, although some *S. cerevisiae* strains can secrete intracellular α-galactosidase, encoded by the *MEL1* gene. These *S. cerevisiae* strains can thus utilize melibiose as a sole carbon source [69,70]. In *S. cerevisiae* and *K. lactis*, galactose is catabolized through the Leloir pathway, which includes a four-step enzymatic reaction [64,65]. In *S. cerevisiae*, the galactokinase Gal1 mediates the initial phosphorylation of galactose in an ATP-dependent manner, resulting in galactose-1-phosphate. Then, uridine diphosphoglucose 4-epimerase (Gal10) exchanges glucose in UDP-glucose with the phosphorylated galactose, thereby generating UDP-galactose, changing the stereochemistry at C4 to create UDP-glucose. In the third step, galactose-1-phosphate uridylyltransferase (Gal7) uses glucose moieties released in the previous step to generate glucose-1-phosphate from galactose-1-phosphate. In the fourth step, glucose-1-phosphate is converted into glucose-6-phosphate through phosphoglucomutase (Gal5) activity. In *S. cerevisiae*, phosphoglucomutase is encoded by *PGM2* (Table 2) [65,71,72,73]. Among the four catalytic enzymes required for the Leloir pathway, Gal1, Gal7, and Gal10, which form a cluster of similarly regulated genes located on chromosome II, are specific to this pathway, while phosphoglucomutase contributes to metabolic pathways for many different carbon sources. 

### 2.2. GAL Gene Regulation in S. cerevisiae and K. lactis

In yeast, the expression of *GAL* genes is tightly regulated by different carbon sources through three main *GAL*-specific regulatory proteins: a transcriptional activator, Gal4; a repressor, Gal80; and a ligand sensor, Gal3 (Table 2). Furthermore, *GAL* regulation is divided into three states depending on the carbon source. First, in the presence of aerobic or respiratory carbon sources, such as glycerol or raffinose, *GAL* genes are in an inactive or non-induced state that is expressed only at basal levels. In *K. lactis*, basal *GAL* gene expression is higher than that in *S. cerevisiae* due to the higher endogenous levels of KlGal4 protein induced by an autoregulatory positive feedback loop [77,78,79,80]. 

In the second state, when galactose is present in the medium, *GAL* genes are in the induced or activated state. In this state, galactose is imported into the cytosol by Gal2 permease, where it binds Gal3p, which also allosterically binds ATP, resulting in Gal3p activation. The activated galactose-ATP-Gal3 complex interacts with Gal80 through a cooperative network of hydrogen bonds [76,81]. As a result of this interaction, Gal80 disassociates from Gal4, and Gal4 is released to transcriptionally activate *GAL* genes, ultimately leading to >1000-fold increases in *GAL2*, *GAL1*, *GAL7*, and *GAL10* expression in *S. cerevisiae* [82]. Apart from *GAL4* itself, the other *GAL* genes harbor specific upstream activating sequences (UAS) in their promoter regions that are recognized by Gal4 homodimers to induce their transcription. Notably, the *GAL4* gene lacks a Gal4 binding site in *S. cerevisiae*, while the *LAC9* gene (i.e., *KlGAL4*) in *K. lactis* has a weak UAS bind site. Consequently, Gal4 concentration in *K. lactis* is two- to three-fold higher than that in *S. cerevisiae* in the non-induced state [74,75]. In addition, there is no *GAL3* gene in *K. lactis*, and its function is substituted by Gal1, a bi-functional protein that acts as a *GAL* gene inducer in addition to its function as a galactokinase [75].

In the third regulatory state, *GAL* gene expression is actively repressed in the presence of glucose, or so-called glucose repression. To induce glucose repression, the concentration of the transcriptional repressor Mig1, a Cys2-His2 Zinc-finger DNA-binding protein, increases within minutes of yeast cell exposure to glucose, after which Gal80 enters the nucleus and interacts with the general co-repressor complex Cyc8/Ssn6-Tup1, to form a complex that targets a specific upstream repression sequence (URSG) present in the promoter region of *GAL* genes [83,84,85]. For *K. lactis*, which is more adapted to lactose/galactose-rich environments, glucose repression of *GAL*/*LAC* gene expression is absent in some *K. lactis* strains, and in strains that do exhibit glucose repression, the repression is less pronounced than in *S. cerevisiae*. This phenomenon is reflected by the fact that only the *GAL1* gene promoter in *K. lactis* carries a URSG, whereas the promoters of *GAL1*/*2*/*3*/*4* all harbor URSG motifs in *S. cerevisiae* [78,80]. 

In addition to this canonical model of *GAL* gene regulation, previously unrecognized regulators of *GAL* gene function are still emerging. For instance, deletion of the cytochrome c oxidase subunit *COX9* results in a respiration-deficient strain that can rapidly and efficiently ferment galactose [86]. Other work has shown that the *SIP1* gene, which encodes a component of the Snf1 kinase heterotrimer complex, is a regulator of preferential glucose consumption in *S. cerevisiae* [87]. Deletion of *SIP1* can destroy the glucose repression at 1:10 ratio of galactose-to-glucose [88]. In addition, the MIG1-related protein Imp2p has also been reported to control *GAL* gene expression by positively affecting glucose de-repression of maltose, galactose, and raffinose metabolic pathways. This protein was also found to contribute to thermal, oxidative, or osmotic stress resistances in yeast [84].

## 3. Bioethanol Production from Whey/Lactose by *Kluyveromyces*

While the fermentation of alcohol from cheese whey or whey permeate represents a relatively new approach to mitigating waste from the dairy industry, yeast-based fermentation of lactose from whey to generate ethanol is first introduced as early as the 1940s, or possibly earlier [89,90,91]. The separation of whey proteins generates whey permeate, which contains the majority of lactose and other whey solids. Industrial facilities that use whey permeate for ethanol production have been established in Ireland, New Zealand, the United States, and Germany. Among these, the first plant to commercially produce ethanol from whey permeate was built in 1978 in Ireland by Carbery Milk Products Ltd. to produce alcohol for beverages. This company has also produced ethanol from whey for E85 and E5 oil blends since 2005 [92]. The process first established at this plant is also employed at fermentation facilities in the United States and New Zealand.

The private producer of ethanol from casein whey feedstock is Anchor Ethanol Ltd., a subsidiary of the Fonterra New Zealand dairy cooperative, which claims to produce ~5 million gallons of ethanol annually. In the United States, whey permeate is fermented at two industrial-scale plants, both using the Carbery process, that are together responsible for 8 million gallons of fuel ethanol annually [93]. The Carbery process uses batch fermentations and continuous distillation in which the pH of the permeate is reduced to 5.0 with sour whey or acid, then pasteurized by heating to 85 °C for 15 s. The pasteurized permeate is then chilled to 34 °C, loaded into the bioreactor, and inoculated with *K. marxianus*. The conditions have been optimized for efficient and rapid lactose conversion to ethanol in 12 h fermentations, with an additional 6 h of cooling in the chamber before distillation [94]. More recently, productivity has been increased through continuous fermentation and use of whey concentrate as feedstock.

Now, the *Kluyveromyces* yeast can mediate lactose fermentation into ethanol because they harbor genes for both lactose permease and β-galactosidase. Among these species, the physiological and molecular characteristics of *K. lactis* have been widely studied as a model for “non-conventional yeasts” in comparative analyses with *S. cerevisiae* [95,96]. *K. lactis* has been used as a progenitor for other strains due to its ability to efficiently utilize the lactose in concentrated cheese whey permeate as a raw material for producing ethanol in static cultures [59]. Apart from applications in ethanol production, *K. lactis* strains are commonly used to produce β-galactosidase [97,98,99,100]. However, this species is generally considered suitable for industrial applications requiring high production and secretion of metabolites or heterologously expressed proteins in a Crabtree-negative dependent manner [101,102,103,104,105,106].

*K. marxianus*, a closely related species to *K. lactis*, has been adopted by several industries due to some useful features that are absent in *K. lactis* [107,108]. Like *S. cerevisae*, *K. marxianus* is a respiro-fermentative yeast that can generate energy either via oxidative phosphorylation and the TCA cycle or by fermentation to ethanol. Thus, *K. marixianus* is frequently used for ethanol production. Several studies have sought to optimize the utilization of lactose in deproteinized whey, cheese whey powder (CWP), cheese whey permeate, and cheese whey in batch and/or continuous mode fermentations [109,110,111,112,113,114,115]. In addition, *K. marixianus* can grow and ferment at elevated temperatures, enabling cost savings in ethanol production bioprocesses [116,117]. Notably, some strains of *K. marixianus* are reported to be highly thermotolerant, growing at 43 °C under aerobic conditions with lactose and/or whey permeate as the sole carbon source [116,118]. However, *K. marxianus* is characteristically Crabtree-negative, and its ethanol yields are typically lower than those of *S. cerevisiae* [118,119].

## 4. Strategies for Conferring Lactose Utilization to *S. cerevisiae*

*S. cerevisiae* is usually the first choice for industrial processes involving alcoholic fermentation due to its high fermentative capacity and ethanol tolerance [120,121,122], GRAS status, rapid growth under anaerobic conditions [123,124], well-established physiology and genetics in industrial and laboratory applications [125], and the value of its biomass as an animal feed [126]. However, wild or wild-type *S. cerevisiae* strains are unable to metabolize lactose, and thus numerous strategies have been devised and tested for endowing *S. cerevisiae* with the capacity for lactose hydrolysis to produce ethanol from whey or cheese whey.

### 4.1. Pre-Hydrolysis of Extracellular Lactose for S. cerevisiae Utilization

Lactose hydrolysis in whey fermentation results in a mixture of glucose and galactose, which can then be taken up by *S. cerevisiae* and fermented into ethanol. Three approaches to extracellular lactose hydrolysis have been established:

First, β-galactosidase can be used to hydrolyze lactose into galactose and glucose, which can then be utilized for ethanol production [127]. However, the presence of glucose induces feedback repression of galactose utilization in *S. cerevisiae*, resulting in assimilation of the produced ethanol, lower biomass, and diauxic growth.

Second, β-galactosidase and/or *S. cerevisiae* cells can be immobilized to produce ethanol [128,129,130]. Through immobilization, the cell densities are higher and thus easier to clear from the medium, thereby reducing the cost of removing cells before distillation. Immobilization can also facilitate simplification of the fermentation process, thus further saving equipment and operating costs. Similarly, strains of *S. cerevisiae* have been developed to ferment a wider range of substrates for combined fermentations, such as whey mixed with cellulosic biomass, by engineering the expression of recombinant cellulolytic proteins anchored or immobilized on the extracellular surface of the plasma membrane along with β-galactosidase [131]. Substantial research efforts have also been committed to developing the substrate for enzyme or cell immobilization and surfactants for improving enzymatic activity, such as silicon dioxide-based nanoparticles, magnetic polysiloxane–polyvinyl alcohol (mPOS–PVA), polymeric supports, and Triton X-100 [132,133,134]. However, immobilization strategies are accompanied by some disadvantages, especially catabolite repression by glucose after lactose hydrolysis and subsequent diauxic growth of the yeast. This lag in lactose fermentation following glucose depletion extends the fermentation time and increases the cost of fermentation.

Third, co-immobilization or co-culture of *S. cerevisiae* with other microorganisms, which secrete extracellular β-galactosidase, can facilitate lactose fermentation by *S. cerevisiae*, thus enhancing ethanol yields and shortening fermentation time. Co-immobilization of the two yeasts has been demonstrated to increase the percentage of theoretical yield over that of monocultures and enhance the overall volumetric productivity. In addition, immobilized co-cultures are reportedly more effective than suspension cultures for high-temperature ethanol fermentations [135,136,137,138,139,140].

### 4.2. Protoplast Fusions of S. cerevisiae and Kluyveromyces *spp.*

Protoplast fusion, a part of evolutionary engineering, has a great potential for genetic analysis and strain improvement. It breaks down the barriers to genetic exchange imposed by conventional mating systems. It can serve the purpose of developing a strain with mixed substrate fermentation abilities [120,141,142]. This technique is generally applied for developing inter specific, intra specific and inter generic, intra generic supra hybrids with higher capability. It is a significant tool for genetic manipulation as it resolves the barrier to genetic exchange imposed by conventional mating systems. It is particularly useful for industrially important microorganisms [142]. Although *S. cerevisiae* cannot utilize lactose, it is tolerant of ethanol and exhibits higher productivity than other fermentation species, making it the most reliable option for ethanol production. In order to enhance production by combining the characteristics of ethanol tolerance and lactose utilization in a single strain, hybrid strains of *S. cerevisiae* and *Kluyveromyces* spp. can be generated through protoplast fusion [143,144,145]. For example, Guo et al. obtained a stable hybrid of *K. marxianus* with an *S. cerevisiae*, showing higher lactose utilization rates and ethanol productivity than the parent strain [144]. Krishnamoorthy and colleagues constructed a hybrid using a temperature-tolerant *S. cerevisiae* with *K. marxianus* that resulted in a 12.5% increase in ethanol productivity at 42 °C [143]. Similarly, Xin generated an *S. cerevisiae*–*K. marxianus* fusion strain with higher lactose fermentation rates and ethanol tolerance than that of the *K. marxianus* parental strain, most likely due to its elevated production of linoleic acid and other unsaturated fatty acids [146]. It should be noted that hybridization can increase the amount of chromosomal DNA in each cell [143,144].

### 4.3. Exogenous Expression of the Lactose Hydrolase Gene in S. cerevisiae

One robust approach for increasing the range of sugar substrates for *S. cerevisiae* fermentation is through the transgenic expression of lactose hydrolysis enzymes from other lactose-consuming microbes. *S. cerevisiae* can thus be metabolically engineered to consume lactose through amplification, cloning, and the introduction of naturally occurring genes or pathways that mediate its uptake and catabolism. The majority of lactose utilization genes used in yeast have been obtained from either *Escherichia coli*, *Kluyveromyces* species, or the filamentous fungus *A. niger*.

#### 4.3.1. Lactose Metabolism Genes from *E. coli*

In *E. coli*, three genes are required for the uptake and metabolism of lactose and related sugars, including *lacZ*, *lacY*, and *lacA* [147,148,149]. *lacZ* encodes β-galactosidase, which converts lactose into allolactose and subsequent metabolic intermediates. *LacY* encodes the lactose permease (LacY) transporter, which mediates lactose uptake into the cell. In order to ensure that *S. cerevisiae*, a eukaryote, can successfully express the prokaryotic *lacZ*, the yeast promoter *CYC1* is typically fused to *lacZ* prior to its transformation into yeast. Since *lacZ* expression is repressed by glucose, an additional ~300 nucleotide DNA fragment is also fused to the expression construct upstream of *CYC1* to drive β-galactosidase expression in the presence of glucose in *S. cerevisiae* [150]. However, although *lacZ* expression was not repressed by glucose in these yeast transformants harboring *CYC1-lacZ*, they were still unable to utilize lactose because they lacked the *E. coli* lactose transport system and therefore could not import lactose to the cytosol for access by β-galactosidase.

To overcome this obstacle, researchers sought to engineer the secretion of β-galactosidase into the culture medium. Porro and his coworkers constructed a lactose-consuming *S. cerevisiae* strain that overexpressed the *lacZ* gene from *E. coli* and then relied on lysis of the older mother cells to release the recombinant β-galactosidase. Cell lysis was induced by overexpression of the transcriptional activator *GAL4*. Characterization of the fermentation properties of the transformed yeast strains indicated that β-galactosidase released in cell lysates enabled growth on lactose as a sole carbon source or in medium containing whey as growth substrate. Furthermore, these strains were found to efficiently produce ethanol during the stationary culture phase following growth on lactose medium [151]. Martegani and coworkers found that cell lysis was independent of heterologous *LacZ* expression in the mother cells [152]. As suggested above, *GAL4* overexpression was proposed as the causative factor in mother cell lysis since the high accumulation of Gal4p can alter regulatory pathways that affect the composition and structural integrity of cell walls, ultimately leading to lysis in older cells. In addition, Gal4p participates in the repression of galactose utilization in the presence of glucose, and excess Gal4p levels have been shown to possibly mediate concurrent glucose and galactose consumption in the *lacZ*-expressing yeast.

#### 4.3.2. The Lactose Metabolism Genes from *A. niger*

*A. niger* can utilize an exceptionally broad spectrum of carbon substrates and is known to secrete numerous glycoproteins, including β-galactosidase, which enables hydrolysis of lactose in acid whey [153]. A lactose-consuming *S. cerevisiae* strain was constructed through the expression of *A. niger* cDNA encoding secreted β-galactosidase. The *lacA* gene (which encodes β-galactosidase in *A. niger*), including its N-terminal signal sequence that directed its extracellular secretion, was inserted between the alcohol dehydrogenase (*ADH1*) promoter and a transcriptional terminator to construct the pVK1.1 vector construct [154]. The pVK1.1 plasmid was then transformed into *S. cerevisiae* resulting in β-galactosidase overexpression. Although the transformant could hydrolyze lactose, the generation time was 8 h in lactose minimal medium. However, the plasmid exhibited high stability, and 84% of the cells still contained the plasmid after nine doublings on whey permeate medium [154]. Ramakrishnan studied the fermentation properties of the transformants, GRF167(pVK1.1). The transformant can hydrolyze the lactose in the medium, and the glucose and galactose from lactose can be utilized at the same time [155]. In addition, a polyploidy distiller’s yeast transformed the same plasmid to obtain a lactose-consuming strain, and the transformants display a higher lactose hydrolysis rate with a simultaneous uptake of glucose and galactose. In addition, the ethanol yield of the transformants is 90.16% the theoretical yield in lactose YP medium; however, the stability of the plasmid is poor, as, during fermentation, only about 10% of the cells retain the plasmid [155].

A lactose-consuming flocculent brewer’s yeast, W204-FLO1L(INT)/pLD1, was constructed through heterologous expression of *lacA* on the pVK1.1 plasmid, the *CUP1* copper resistance marker used to screen successful transformants. The transgenic yeast cells were able to use lactose with no obvious impact on flocculability [156]. Domingues and coworkers used a flocculent uracil auxotrophy mutant of *S. cerevisiae* carrying pVK1.1 as a parent strain to construct a flocculent lactose-consuming strain, NCYC869-A3/pVK1.1 [157]. The recombinant yeast exhibited high extracellular β-galactosidase activity. Although galactose uptake was at least partially repressed by glucose in this strain, the strain showed a lactose uptake rate close to 1.7 g/L/h and with ethanol contents close to the maximum theoretical yield [157].

#### 4.3.3. The Lactose Metabolism Genes from *Kluyveromyces* spp.

Different from *A. niger*, which breaks down lactose in the extracellular space, yeast in the genus *Kluyveromyces* transports lactose into the cell for metabolism (Figure 2b). Therefore, a lactose permease is needed to access the substrate in addition to the lactose hydrolase. The lactose hydrolase, i.e., β-galactosidase, is encoded by the *LAC4* gene, while the lactose permease is encoded by the *LAC12* gene. Since *Kluyveromyces* is relatively close to *S. cerevisiae*, phylogenetically, the eukaryotic lactose transport system from *Kluyveromyces* is more compatible with *S. cerevisiae* regulatory mechanisms. Thus, *S. cerevisiae* can be engineered to grow on lactose through the simultaneous expression of lactose permease and non-secreted β-galactosidase from *Kluyveromyces*.

Sreekrishna was the first to construct Lac+ *S. cerevisiae* strains using a shuttle vector carrying a 13 kb region of the *K. lactis* genome [158]. The 13 kb region contained *LAC4* and a flanking upstream sequence from *K. lactis*. Despite the low lactose uptake rate in transformants, these uptake assays confirmed that the lactose permease gene, *LAC12*, was contained in that flanking sequence from *K. lactis*, between 2.0 and 8.6 kb upstream of *LAC4*. That study represented the first report of heterologous expression of a eukaryotic membrane-bound permease, which laid a foundation for the subsequent cloning of lactose permease and lactose hydrolase genes from *K. lactis* [158]. In order to improve the lactose uptake rate and increase the mitotic stability of the heterologous construct, researchers then created the MRY276 yeast strain by inserting the *LAC4* and *LAC12* genes from *K. lactis*, under the control of the *CYC1-GAL* galactose-inducible promoter, into the *RDN1* (ribosomal DNA) locus [159]. MRY276, which harbored multiple copies of both *LAC4* and *LAC12*, retained the Lac+ phenotype for longer than 60 generations in growth on non-selective medium, although it exhibited slow growth. The suboptimal growth characteristics of this strain were likely attributable to the burden imposed by overexpression of these transgenes. To resolve this issue, transgenic MRY276 was crossed with wild-type strains to yield meiotic segregants with faster growth and the capacity for lactose assimilation, such as the diploid strain MRY286 [159]. However, this strain showed low ethanol production but high biomass production on lactose minimal medium.

Domingues constructed the Lac+ flocculent *S. cerevisiae* strain, T1, that expressed *LAC4* and *LAC12* from *K. marxianus* rather than *K. lactis* [160]. The T1 strain showed a lower capacity for flocculation than that of the parent strain but showed a doubling time of 5 h, which was substantially faster than the 6.7 h reported for the original Lac+ strain constructed by Sreekrishna and Dickson [158], but slower than the *K. marxianus* donor of the *LAC4* and *LAC12* genes. However, after an adaptation period, where the strain was kept in liquid lactose medium, refreshed periodically, fermentation assays indicated that T1 could metabolize ~90 g/l lactose in liquid medium, thus highlighting increases in rates of both biomass accumulation and ethanol production from lactose.

In general, the use of a lactose-consuming strain for direct fermentation of whey to produce ethanol is not economically feasible because the low lactose content in whey results in low ethanol titer (i.e., 2–3% *v*/*v*). Therefore, fermentation should begin with concentrated whey to obtain high ethanol yields at the end of the process, which also requires high tolerance to ethanol and osmotic pressure. In *S. cerevisiae*, the accumulation of trehalose is widely considered a critical determinant for the improvement of stress tolerance in yeast, and the deletion of the trehalose degrading enzyme gene can significantly increase intracellular trehalose content [161].

In particular, a neutral cytosolic trehalase (*NTH1*) and an acidic vacuolar trehalase (*ATH1*) have been identified as the main trehalose hydrolases in *S. cerevisiae* [162,163]. In order to obtain a Lac+ *S. cerevisiae* strain that tolerates high sugar and ethanol conditions associated with concentrated whey fermentation, the Δ*ath*Δ*nth* Lac+ strain AY-51024A was generated, which expressed the permease and β-galactosidase genes from *K. marxianus* [164]. In this strain, the *ATH1* and *NTH1* loci were used as the target regions for integrating the *LAC4* and *LAC12* genes driven by the *PGK1* promoter. AY-51024A showed roughly equal β-galactosidase activity during growth on different carbon sources and exhibited high tolerance to ethanol, and could withstand higher osmotic pressure than the parent strain. In addition, it showed an almost equal rate of glucose uptake and a higher rate of galactose uptake than its parent strain, AY-5. However, this transgenic strain was subject to glucose repression, and lactose uptake rates were very slow in lactose medium (0.98 g lactose/g cell/h). Further analysis confirmed that glucose repression of lactose/galactose metabolism was responsible for the slower lactose uptake rather than the result of low copy number of *LAC4* and *LAC12* [164].

## 5. Approaches for Enhancing Lactose Utilization Rate

Considerable efforts have been undertaken to increase lactose uptake rates in order to improve the efficiency, and hence economic viability, of whey, lactose, or cheese whey as substrates for bioethanol production. These efforts largely follow three approaches, discussed below.

### 5.1. Optimizing Culture Conditions and Media Components

While selecting or developing a strain with appropriate metabolic capabilities may be an essential step in bioprocessing, low yield rates from lactose to ethanol and ethanol production rates pose the largest obstacle to efficient whey fermentation. The performance of fermentation systems is strongly affected by culture conditions such as temperature, pH, initial inoculum, substrate and nutrient concentrations, trace element availability, and other factors. Therefore, optimizing culture conditions represents an essential step, and often underestimated challenge, in fermenting whey products or lactose to bioethanol. Several approaches have been developed for evaluating and optimizing factors that affect fermentation time and ethanol production, such as response surface methodology (RSM), central composite design (CCD), and orthogonal design [112,127,165,166,167]. In addition, electro-activation has also been used to enhance both biomass and ethanol production [49].

In addition to culture conditions, optimizing media components is also necessary for effective ethanol production. Sufficient availability of nitrogen, phosphorus, trace elements, vitamins, and co-factors, are all important to ensure that fermentation proceeds to completion as fast as metabolically possible. Thus, inexpensive and nutritionally complete inputs are needed to supplement industrial whey fermentation [115]. One widely reported, nutrient-rich input for whey fermentation is corn steep liquor (CSL), which is the primary by-product of corn starch production. CSL contains abundant nutritional resources, such as proteins, amino acids, vitamins, minerals, and trace elements, and is relatively inexpensive and easily obtained [168,169,170]. In fermentations using the recombinant T1-E strain for ethanol production from cheese whey powder solutions (CWPS), the addition of 10 g/L CLS resulted in significantly increased yields of 7.4% (*v*/*v*) ethanol from 150 g/L initial lactose, with a productivity rate of 1.20 g/L/h, whereas 0.75 g/L/h ethanol was obtained in the absence of CLS. Notably, this approach also stimulated lactose uptake [171].

### 5.2. Metabolic Engineering

Since it was first defined as ‘the improvement of cellular activities by manipulation of enzymatic, transport, and regulatory functions of the cell with the use of recombinant DNA technology’ [172], metabolic engineering has been broadly applied to restructure metabolic networks by altering the reaction rates and distribution of (carbon) resources in specific pathways in order to improve metabolite and protein production. As previously mentioned, although *S. cerevisiae* cannot utilize lactose, it can consume the lactose metabolite, galactose. Galactose metabolism is subject to dual control by glucose and *GAL* genes in *S. cerevisiae*. Research has revealed that glucose repression of galactose metabolism affects the overall lactose metabolic process in Lac+ strains [164]. The transcription of *GAL* genes is regulated Gal4, which is repressed by the Ssn6-Tup1-Mig1 complex in the presence of glucose. Mig1, in particular, recognizes a *GAL4* promoter motif, and complex binding to this motif represses *GAL4* transcription. In addition to glucose repression, *GAL* genes are also negatively regulated by Gal80 and Gal6.

Since eliminating glucose repression can improve the rate of galactose catabolism [173,174,175], and the rate of galactose metabolism impacts lactose uptake [164], deletion of *MIG1* in Lac+ *S. cerevisiae*, such as reported in strain AY-51024M, should decoupling the co-regulation of glucose and galactose of glucose repression. The Δ*mig1* strain showed a 2.55-fold increase in the rate of lactose uptake, and ethanol production increased by 1.75-fold compared with the control strain, AY-51024A, which harbored intact *MIG1* gene, in anaerobic shaken flask fermentations of lactose medium. In CWPS medium, AY-51024M could produce 63.3 g/L ethanol from a 150 g/L lactose starting concentration in 120 h fermentations. By contrast, under glucose repression, AY-51024A produced only 35.9 g/L ethanol, consuming 63.7% of the lactose input [164].

Apart from glucose repression, the regulation of *GAL* genes also impacts the rate of galactose uptake. For example, the deletion of *GAL6*, *GAL80*, and *MIG1*, which all downregulate *GAL* genes, can increase galactose uptake rates by 41% while overexpressing the transcriptional activator *GAL4* increases uptake rates by 26% [174]. Other studies reported similar effects of manipulating *GAL* regulators [174,175]. Rønnow and colleagues showed that deletion of *MIG1* and *GAL80*, combined with transgenic expression of *MEL1*, which encoded melibiase, resulted in a melibiose-consuming strain that can metabolize galactose, even in the presence of glucose. This strain could produce ethanol through the utilization of melibiose in molasses substrate [176].

Similarly based on the deletion of *MIG1*, the Δ*mig1*Δ*gal80* Lac+ *S. cerevisiae* strain AY-GM, Δ*mig1*Δ*gal6* strain AYG6M, and Δ*gal6*Δ*gal80*Δ*mig1* strain AYG68M were all constructed using the *loxp-KanMX-loxp* resistance cassette [177]. However, AYG6M and AYG68M performed the same or significantly worse than AY-GM and/or AY-51024M in preliminary assays for carbon uptake and utilization, stress tolerance, and ethanol production [177]. Compared with the Δ*mig1* strain, AY-51024M, diauxic growth and glucose repression were both abolished in AY-GM, leading to significantly higher galactose uptake rates and final ethanol concentrations. Moreover, tests of AY-GM in three repeated 5L batches using CWPS medium with 100 or 150 g/L lactose revealed significantly higher rates of lactose uptake and ethanol production compared to AY-51024M, with lower fermentation times.

### 5.3. Evolutionary Engineering

In contrast with metabolic engineering, directed evolution or evolutionary engineering is another main strategy for obtaining high-performance strains for industrial ethanol fermentation. Evolutionary engineering can be used as an alternative to metabolic engineering strategies to circumvent obstacles in cloning and gene expression that result from the incomplete characterization of metabolic networks or a lack of sufficient genetic/metabolic information about strains of interest. Directed evolution-based strategies thus represent an inverse approach to that of metabolic engineering in that they can be used to generate diversity or explore mechanistic differences in performance as a starting point for metabolic engineering. In *S. cerevisiae*, evolutionary engineering is generally applied (1) to modify substrate utilization and product formation or (2) to alter stress resistance [178,179,180,181].

Thus, evolutionary engineering can be used to generate lactose-consuming *S. cerevisiae* strains. For example, this strategy was used to generate the T1 strain, a Lac+ strain that can grow on lactose but slowly [160]. Guimarães and coworkers subsequently built on this work, using evolutionary engineering to obtain a highly effective lactose-utilizing *S. cerevisiae* strain, T1-E [182]. After batch selection by serial dilution of cells and transfer to medium with gradually increasing lactose content, the evolved T1-E exhibited two-fold higher rates of lactose consumption, 30% greater ethanol production, and the ability to efficiently ferment concentrated cheese whey [182]. These effects on improved lactose fermentation were the result of two molecular events. First, the plasmid copy number was decreased by 10-fold that of T1, and second, a deletion was introduced in the intergenic region between *LAC4* and *LAC12* [182]. Further transcriptomic analysis revealed that the expression levels of 173 genes differed by more than 1.5-fold in the engineered strain, roughly half of which were related to RNA-mediated transposition [183]. Although evolutionary engineering requires more time and does not show the molecular mechanism underlying new phenotypes, which are disadvantages compared to metabolic engineering, evolution-based strategies can be an efficient means of inducing metabolism of unfavorable carbon sources.

## 6. The Future of Ethanol Production from Whey or Lactose by Yeast

Given the high abundance of organic compounds in whey or cheese whey, combined with the increasing amounts of these by-products generated by dairy production facilities, the utilization or disposal of whey and cheese-whey pose a non-trivial problem as well as a threat to the ecosystem [34,184,185]. In future solutions to this issue, whey utilization may be categorized by application. From the perspective of maximizing the value of this industrial by-product, whey could be used in several different fields, such as the food industry [184,186,187,188], animal or livestock feed production [186,189,190], as a raw substrate for industrial biosynthesis of recombinant proteins, lactic acid bacteria culture, or for synthesizing metabolites such as L (+) lactic acid and medium-chain carboxylic acids [191,192,193,194], or in the production of alcoholic beverages [195,196,197], or whey-based fruit-flavored beverages [198,199,200]. Alternatively, from the perspective of solving the feedstock shortage for bioethanol, future research will likely focus on whey-based raw materials and development of efficient strains.

### 6.1. Whey/Cheese Whey Mixed with Other High Carbon Feedstocks for Bioethanol

As the lactose content is relatively low, direct fermentation of whey/cheese whey to ethanol is generally not economically viable and results in low ethanol titre (2–3% *v*/*v*), making distillation costs prohibitively expensive. Thus, concentrated whey powder (CWP) represents an effective option for fermentation but requires yeast with a high tolerance for lactose and ethanol. Alternatively, the sugar concentration can be increased by mixing native whey with other easily-obtained or sustainable materials. Lignocellulosic feedstock is considered the primary input material for second-generation bioethanol [201]. Lignocellulosic plant matter is the most abundant form of biomass for ethanol fermentation and can thus serve as a sustainable and cost-effective substrate for bioethanol production. Approximately 650 billion tonnes of carbon in lignocellulose are estimated to be stored in forests worldwide, and the forestry or timber industries generate staggering amounts of sawdust and thinned trees, representing a completely unused lignocellulose resource, accompanied by increased greenhouse gas emissions. Among harvested trees, *Eucalyptus* is the most widely planted hardwood (approximately 18 million ha) and includes over 700 species across tropical, subtropical, and temperate growing regions. Therefore, *Eucalyptus* waste is a strong candidate raw material for bioethanol production [202,203]. In order to use these wood by-products for fermentation, they are first subjected to autohydrolysis or hydrothermal treatment in hot water. This process is purported to have low environmental impacts and enables simultaneous saccharification and fermentation (SSF) of bioethanol from lignocellulose in hardwoods or agro-industrial waste. In order to increase sugar concentration, the hydrothermally pretreated *E. globulus* wood (EGW) can also be mixed with cheese whey powder to increase the available carbon and subsequently obtain high ethanol concentrations [42]. Whey can also be mixed with other cellulosic biomass than *E. globulus*, such as sugarcane bagasse [204,205], spent brewer’s grains [206], or corn cob [131], all of which improve ethanol yields and result in high ethanol titers when mixed with whey to increase nitrogen and micronutrient bioavailability.

### 6.2. Approaches for Efficient Generation of Lactose-Consuming Strains

Obtaining robust yeast strains with strong industrial potential is equally important for economical bioethanol production as using a high-concentration carbon source to intensify the ethanol titre. In particular, high-performance yeast should exhibit rapid conversion of sugar substrate to ethanol, as well as tolerance to high ethanol concentrations and high temperatures. Advances in metabolic, evolutionary, and genetic engineering have facilitated the development of research methods for improving lactose/galactose metabolism. For instance, Chunha used genetic engineering to construct a *S. cerevisae* strain that can simultaneously utilize the sugars from cheese whey powder and a zymolytic solution of hydrothermally treated *E. globulus* in a multi-waste valorization [42]. In another good example of inverse metabolic engineering, Lee and coworkers screened a genome-wide perturbation library to identify three galactose utilization-enhancing genes, including the phosphomannomutase, *SEC53*, a small nuclear RNA, *SNR84*, and a general repressor of transcription, *TUP1* [207]. While *SNR84* overexpression improved both growth and ethanol production from galactose, the overexpression of a truncated *TUP1* variant, *tTUP1*, resulted in strikingly increased capability for galactose fermentation, 250% higher in both the rate of galactose consumption and ethanol productivity compared to that of the control strain. In addition, overexpression of *tTUP1* significantly shortened the lag periods accompanying the change in carbon source from glucose to galactose [207]. The mechanism for improving galactose metabolism through *SEC53* overexpression may be similar to that of *PGM2* overexpression, which encodes a phosphoglucomutase [208].

Adaptive evolution may also serve as an effective strategy for improving galactose metabolism, especially in combination with metabolic engineering, as illustrated in the following examples. In a screen for unique strategies for improving galactose uptake in yeast, Hong et al. used integrated, systems-level analyses to determine the mechanisms responsible for enhancing galactose metabolism over ≈400 generations of serial culture on galactose [209]. They found that isolates that evolved the most efficient galactose consumption harbored mutations in Ras/PKA pathway proteins, which mediate global carbon sensing, and that these strains had upregulated reserve carbohydrate metabolism and ergosterol biosynthesis. In particular, the *RAS2^Tyr112^* mutation resulted in a higher specific growth rate on galactose, indicating that adaptive evolution can lead to increased galactose flux through cellular exploitation of unexpected pathways, whereas rationally engineered strains rely on pathways with known effects. This demonstration of the effectiveness of adaptive evolution suggests its potential as a viable alternative approach to bioengineering metabolically-enhanced strains compared to rational design. In conjunction with systems biology analyses, genes harboring mutations that strongly affect metabolism can be rapidly identified and used as targets of further metabolic engineering to enhance microbial production of biofuels and other valuable metabolites [209].

## 7. Conclusions

The research focused on using biodegradable wastes, such as whey, for ethanol production by combining biochemical pathways from various organisms is very important for both reducing environmental impacts of food production while improving, cost-effectiveness and sustainability for other industrial sectors (such as commercial polymers and fuel production). Despite the numerous innovations described here, the potential for renewable energy production and other applications of whey fermentation are largely untapped. Thus, the research aimed at improving production efficiency and energy consumption through fermentation of agricultural by-products such as cheese whey will provide substantial economic benefits while decreasing energy dependence and mitigating the effects of pollution as developing nations adopt these advanced technologies.

## Figures and Tables

**Table 1:** Composition of different types of whey [27,28].

Parameter	Sweet Whey	Acid Whey
Total solid (%)	6.21	5.70
Lactose (%)	4.82	4.60
Protein (%)	0.75	0.30
Fat (%)	0.05	<0.01
Ash (%)	0.60	0.80
pH	5.80–6.10	4.0–5.0

**Table 2:** Galactose/lactose catabolic and regulatory genes and their respective annotations in *S. cerevisiae* and *K. lactis*.

Category	*S. cerevisiae*		*K. lactis*	
	Gene Name	Function	Gene Name	Function
Catabolic genes	*MEL1* ^1^	α-galactosidase	*LAC4*	β-galactosidase
	*GAL2*	Galactose permease	*LAC12*	Lactose/galactose permease
	*GAL1*	Bifunctional galactokinase/sensor	*KlGAL1*	Bifunctional galactokinase/sensor inducer [74,75]
	*GAL7*	Galactose-1-phosphate uridylyltransferase	*KlGAL7*	Galactose-1-phosphate uridylyltransferase
	*GAL10*	Uridine diphoshpoglucose 4-epimerase	*KlGAL10*	Uridine diphoshpoglucose 4-epimerase
	*GAL5(PGM2)*	Phosphoglucomutase	*KlGAL5*	Phosphoglucomutase
Regulatory genes	*GAL4*	Transcriptional activator [75]	*KlGAL4(LAC9)*	Transcriptional activator [74,75]
	*GAL80*	Gal4p repressor [74,75]	*KlGAL80*	Gal4p repressor
	*GAL3*	Gal80 repressor (sensor/inducer) [76]		

^1^. Only some strains of *S. cerevisiae* carry the *MEL1* gene.

## Data Availability

Not applicable.
